# Vacuum Seed Sowing Manifold: a novel device for high-throughput sowing of Arabidopsis seeds

**DOI:** 10.1186/1746-4811-9-41

**Published:** 2013-10-22

**Authors:** Richard Barker, Benjamin Cox, Thomas Rockwell Mackie, Patrick Masson

**Affiliations:** 1 Laboratory of Genetics, Agriculture and life sciences, University of Wisconsin at Madison, 3302 Genetic/Biotech center, 425 C Henry Mall, Madison, WI 53706, USA; 2 Medical Devices Group, Morgridge Institute for Research, 330 North Orchard St, Madison, WI 53715, USA; 3 Department of Medical Physics, University of Wisconsin at Madison, 1111 Highland Avenue, Room 1005, Madison, WI 53705, USA

**Keywords:** Arabidopsis, High-throughput, Seed-sowing, VSSM, Novel device

## Abstract

The small size of Arabidopsis provides both opportunities and difficulties for laboratory research. Large numbers of plants can be grown in a relatively small area making it easy to observe and investigate interesting phenotypes. Conversely, their small size can also make it difficult to obtain large quantities of tissue for investigation using modern molecular techniques. Sowing large numbers of their seed can overcome this; however, their small seed size makes this difficult. Here we present the Vacuum Seed Sowing Manifold (VSSM), a simple device that can be printed using a 3D printer and provides a new high throughput method to sow large numbers of seeds at a range of densities.

## Background

*Arabidopsis thaliana* was the first higher organism to have its genome sequenced and is now widely regarded as the model dicot. This led to the production of a vast amount of information being made publically available, and sped up research on this and other similar species [1,2]. *Arabidopsis* was chosen as a model for many reasons, including its small size, fast life cycle, self-fertile flowers, simple morphology and the ease with which it can be genetically transformed. Since the original sequencing of the Col-0 ecotype a project to sequence another 1000 accessions has begun in order to map its natural diversity [3]. This further increases the number of methods for investigating this organism at a molecular level, which also increases the need for high throughput phenotyping strategies to assess the morphological effect of modifying its genetic code or testing different environmental perturbations [4].

These newly opened fields of research are advantageous to the study of *Arabidopsis*, but they also lead to new obstacles that need to be overcome. In particular, the large number of sequenced accessions and mutant lines means that there are many variations that need to have their phenotypes quantified. The small size of the seeds makes them difficult to sow, but does provide many opportunities, as the resulting seedlings are also small. Their small stature can easily be observed when grown on clear agar-based media, allowing the organs’ growth behavior to be quantified. This is particularly important for roots that cannot easily be observed when grown in soil. In addition, the sterile solid agar media also makes root tissue easily accessible, which can be harvested for DNA, RNA and protein extraction.

The sequenced and well annotated genome makes *Arabidopsis* the perfect plant for the application of new research methods such as RNAseq, smallRNAseq, ChipSEQ and proteomic screens such as iTRAQ [5-7]. Each of these factors puts ever increasing pressure on researchers to sow larger and larger numbers of *Arabidopsis* seeds so that their various tissues can be harvested for further investigation. As an example, an iTRAQ-based proteomic analysis of root gravitropism and mechano response recently developed in our laboratory required analyzing 250 μg of root-tip protein (extracted from approximately 2000 seedlings) per experimental treatment. That is for a total of 8 treatments, implying that over 16,000 seedlings had to be grown and dissected per experimental repeat. Considering that the experimental set up had to be repeated three times, this analysis required germinating and growing a total of approximately 50,000 Arabidopsis seedlings on agar-based medium for subsequent root-tip dissection and protein extraction. Without a method of high-throughput seed sowing, conducting experiments such as these would be impossible.

Despite the vast array of technical applications of *Arabidopsis* research, only relatively simplistic and low throughput methods are currently employed for sowing *Arabidopsis* seeds on the surface of agar plates. Currently, researchers either use a pipette to dispense the seeds in a liquid solution or use a toothpick to carefully place the seeds on the appropriate growth media. Sowing seeds using these methods can be particularly difficult when they must be sown in a straight line on an agar petri dish, which is often the case. This is particularly true for groups with an interest in root morphology. In cases where time and people are in short supply, this can prohibit certain experimental designs that call for a large number of seedlings. Performing these experiments requires the development of alternative seed sowing strategies.

**Figure 1:**
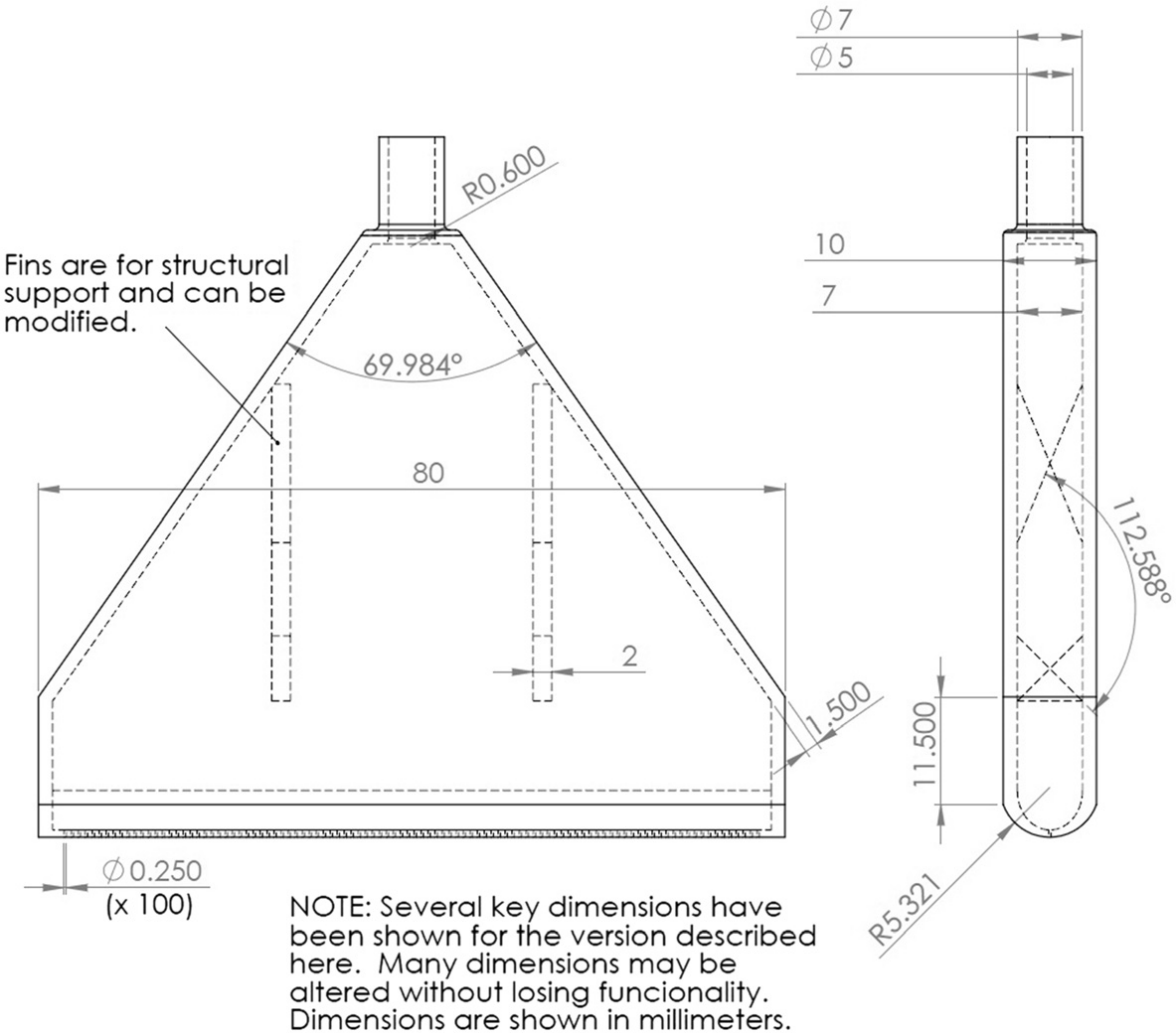
**Computer-aided design (CAD) drawing of the SS manifold.** Units shown are in inches unless otherwise specified. Drawing was made using SolidWorks Software (Dassault Sytemes, Waltham, MA).

**Figure 2:**
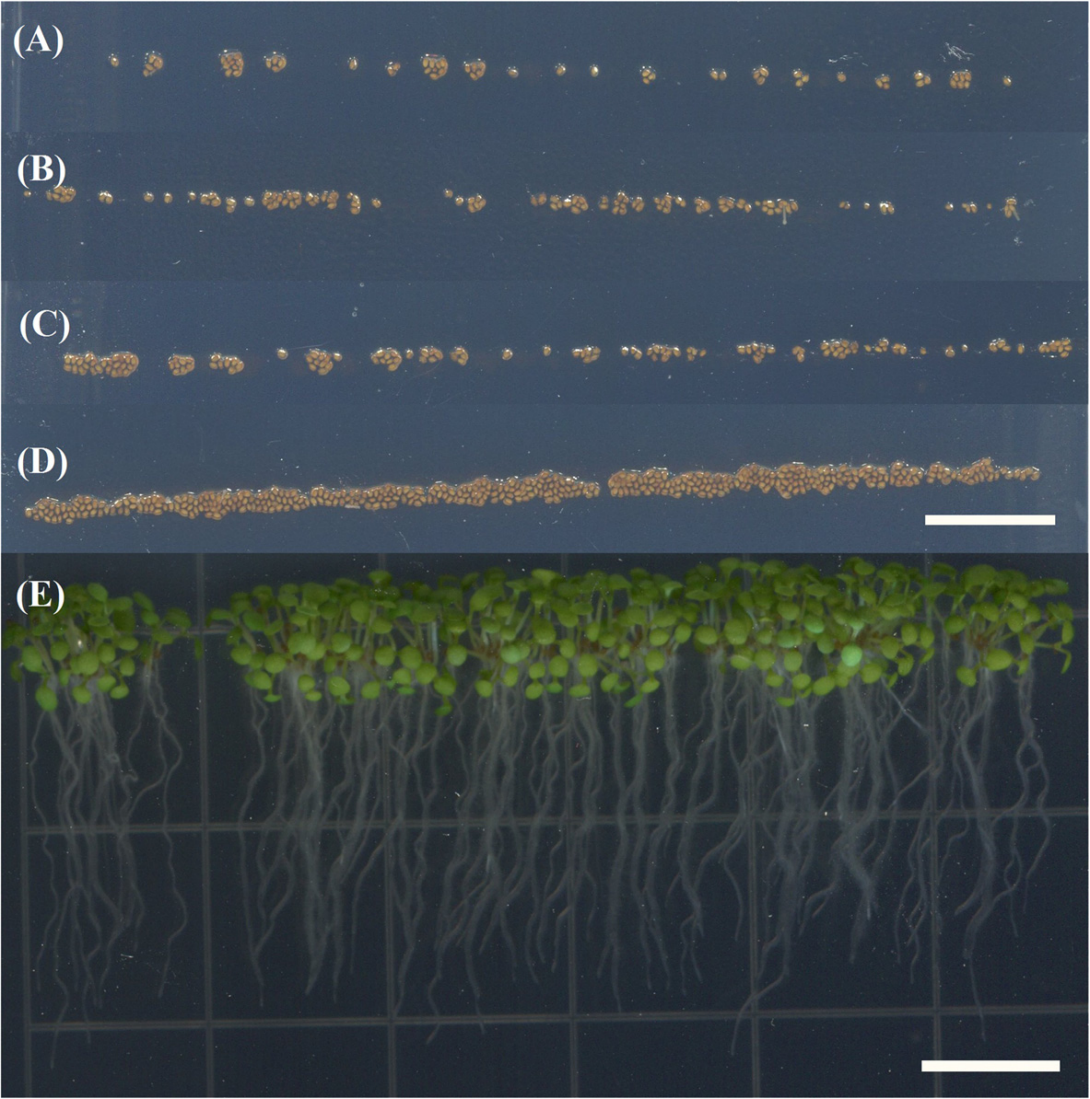
**Example lines of seeds sown using VSSMs with different numbers of holes. (A)** 25 holes. **(B)** 50 holes. **(C)** 75 holes. **(D)** 100 holes. **(E)** Example of a line of seedlings, 7 days after growth, sown with a VSSM with 75 holes. Scale bars shown are 1 cm.

To address this issue we developed the Vacuum Seed Sowing Manifold (VSSM) (Figure 1). This simple device allows a researcher to pick up approximately 100–200 sterilized seeds at once and deposit them on an agar plate in a straight line in less than 30 seconds. The plants can then be grown and seedling tissue harvested for further investigation. Examples of lines of seeds sown using this device are shown in Figure 2.

The Seed Sowing Manifold was manufactured using three-dimensional (3D) printing techniques. 3D printing is the process of creating a part by adding material layer by layer [8]. It is often called ‘additive’ manufacturing to contrast with typical machining methods that are subtractive. Rather than removing material to create a part in additive manufacturing material is continuously added. There are several different 3D printing processes and their development enabled the fabrication of complex geometries [9]. Objects that would have been extremely difficult and maybe even impossible to build are now routinely manufactured. The device described here is an example of a part that would have been extremely hard to fabricate without the use of 3D printing. It was manufactured on a Viper Si2 stereolithography machine (3D Systems, Inc., Rock Hill, SC).

Stereolithography (SL) is a type of 3D printing that solidifies liquid resins in a three-dimensional pattern forming a completed part and is also the oldest type of 3D printing processes [10]. During the SL process, a liquid photopolymer resin is solidified in a precise pattern using UV light, which crosslinks the polymer’s chemical backbone. When a project is begun, the build platform rests at the surface of the resin. A UV laser that is guided by 2 galvanometric mirrors scans the outline of one layer of the part, solidifying it. When one layer is done, the platform submerges slightly to allow for the next layer to be cured. This is repeated until the part is complete.

## Results and discussion

### Description and drawing

The seed sowing manifold consists of one plastic piece. This plastic piece is roughly triangular in shape, and is hollow in the center. The interior cavity dictated the angle that was needed on the sides of the triangle. This was because the cavity was printed without supports inside it, and this is only possible with steep enough angles (see Additional file 1 for more details). There are two fins on the inside of the manifold. These fins are trapezoidal in shape and serve as structural support, preventing breakage along the row of holes by vacuum-driven squeezing stress during the utilization of the device (see Figure 1). Their trapezoidal shape was also dictated by the need to remove supports inside the cavity of the part.

The seed sowing manifold has two functional ends. The top (point of the triangle) is where the vacuum is applied and the bottom (wide end of the triangle) is the end that contacts the seeds, or the planting end. At the planting end, there is a row of 100 holes that are 250 microns in diameter. The size of these holes could be modified for different size seeds. At the top (point of the triangle), there is a tube with an outer diameter of 7 mm and an inner diameter of 5 mm. This tube is meant to be an adapter to the rubber tubing of a vacuum setup. This is an embodiment of the device that worked well for our needs, but most of the dimensions, including height, diameters and spacing of the holes, and wall thickness can be adjusted without loss of functionality.

#### Fabrication

**Figure 3:**
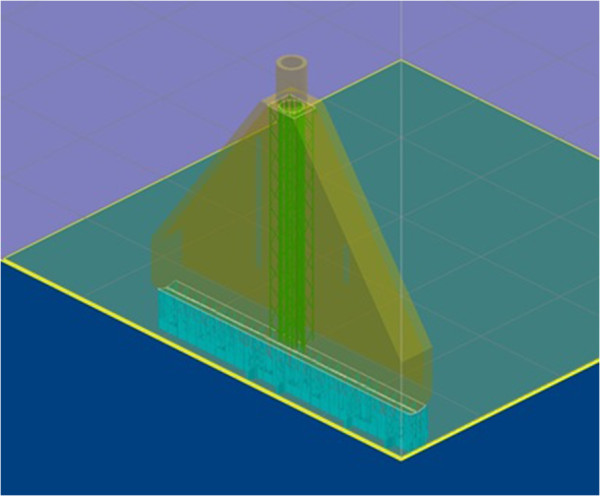
**Screenshot of 3D Systems’ build setup software (3D Lightyear) during the setup of a build with the SSM on it.** The SSM is oriented as described. There are two surfaces that the program placed supports on. The supports that were removed are shown in green.

The VSSM was fabricated using a Viper Si2 SLA system (3D Systems, Inc., Rock Hill, SC). It was printed standing up vertically in the z-direction with the row of 100 small holes facing downward. Looking down on the x-y plane, it was oriented so the long edge ran along the y-axis of the machine. This was because the resin is recoated in the y-direction and orienting this way helps to minimize defects in the part and improve resolution of the holes. Supports were removed from within the hollow void in the part, because they would be very difficult to remove after the fabrication process. Because of this, any angles in the design of the device had to be steep enough that the machine used to fabricate them could do so without including support structures. Figure 3 illustrates this setup.

The VSSM was fabricated out of Accura60 resin. This is a material that is proprietary to 3D Systems, Inc. It is chemically similar to UV-cure epoxy and also requires UV light to solidify. As a liquid, it is very viscous like most SLA resins, but as a solid, it has properties similar to acrylic. Although its chemical formulation is strictly proprietary, the MSDS sheet and datasheet for Accura60 are available from the manufacturer.

#### Seedling yield

**Figure 4:**
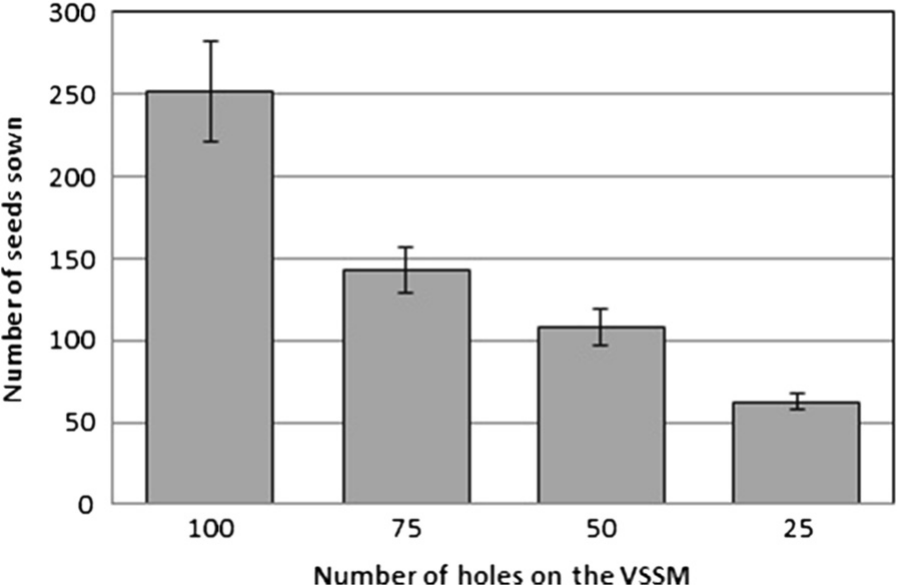
**A comparison of the number of seeds sowed using VSSM with 25, 50, 75 or 100 holes.** 10 agar plates were sown with one line of seeds per VSSM design.

While seedlings are sown in a straight line using this process, the number of seedlings sown is nearly always more than the amount of holes in the VSSM. This is because it is impossible to get all of the seedlings that are not fixed to a hole off the VSSM, without also removing some that are fixed to a hole. In addition, the air flow into a hole may enable more than one seed to adhere near a hole. To quantify the amount of seeds that were actually sown as a function of the number of holes in the VSSM, 4 different versions of the VSSM were constructed with varying amounts of holes (Figure 2). The numbers of holes used were 25, 50, 75, and 100 holes. Each of these 4 versions was used to sow 10 rows of seeds and the average number of seeds actually sown was plotted against number of holes (Figure 4).

#### Fungal contamination

**Figure 5:**
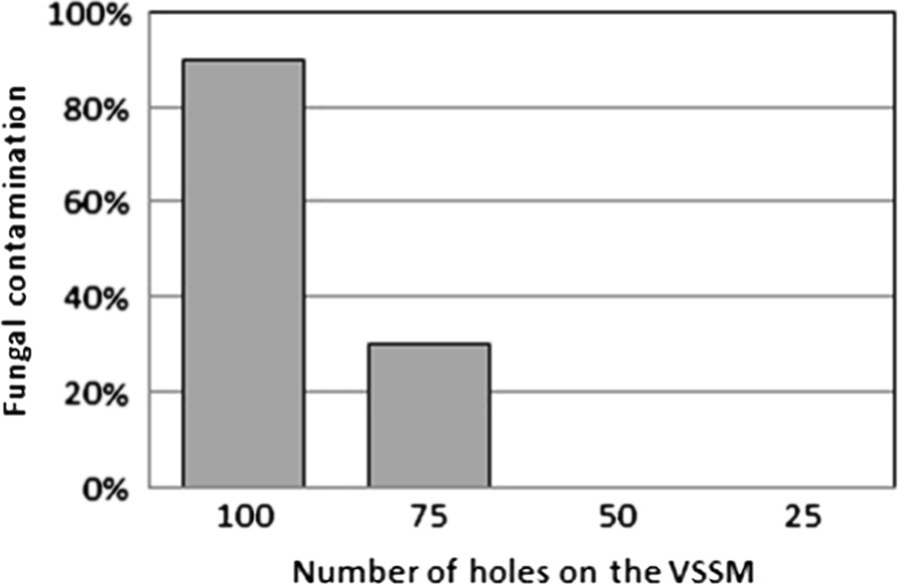
**A comparison of the amount of fungal contamination and the number of seeds sown.** Percentage of plates that got contaminated by fungi reveals that high sowing densities are more likely to get contaminated.

When high densities of seeds were sown, fungal contamination killed many plates. The amount of fungal contamination was examined as a function of the number of holes in the VSSM. The results of this analysis can be seen in Figure 5. It is clear from these data that the highest two densities of seeds examined were susceptible to fungal contamination, with the highest seed density exhibiting the highest contamination.

This is due to the presence of fungal spores amongst the seeds that are planted. While the seeds are sterilized prior to planting, the sterilization is not perfect. Sowing a higher number seeds increases the chance that a seed with fungal spores on it will be present. It is also possible that seed density influences the likelihood of fungal contamination as well. There was heavy contamination observed when the VSSM with 100 holes was used. However, using the VSSM with 50 holes, it was possible to sow 10 plates without any fungal contamination. This suggests a threshold seed density, above which, fungal contamination is a concern.

## Conclusions

Throughout the seed sowing procedure, we use multiple manifold adapters and regularly wash them with EtOH to avoid device clogging and potential contamination. In addition, it is important to note that the line of seeds deposited by the SSM on the agar-based medium is not always perfectly straight and continuous (see Figure 2). The number of seeds per row can vary slightly depending on alterations to the sowing method such as the number of seeds available for grasp on the filter paper. The number of holes in the VSSM will alter the number of seeds it sows (see Figures 2 and 4). If large numbers of seeds are sown in excess of about 150 seeds per plate, then the amount of plates lost to fungal contamination also increases. Thus, we recommend between 25–75 holes to maximize the number of seeds sown while minimizing losses due to contamination.

While the particular design described here met the needs of this lab, other embodiments could work as well. Future work will include the development and testing of designs that would be compatible with other popular platforms. Three that have been discussed include versions for the even dispersal of seeds across a circular petri dish, a square petri dish, and a 96-well plate.

The seed sowing manifold increases the amount of seeds that can be sown by a researcher. This will allow researchers to design larger than previously possible experiments. The number of holes can be varied allowing sowing densities to be controlled according to the experimental parameters or researcher preference. When combined with high throughput phenotyping platforms, it will allow a greater number of mutants and ecotypes to be investigated. It will also increase the amount of tissue (such as root tips) that researchers can harvest, making studies that require large amounts of tissue more viable (such as iTRAQ).

Finally, this is a great example of a problem that was perfectly suited for interdisciplinary efforts. The design, fabrication, and use of this device required varied skill sets and resulted in a useful new research tool. The seed sowing manifold is a result that might not have been possible without this type of collaboration. The computer-aided design (CAD) files for the seed sowing manifold are also available (see Additional file 2).

## Methods

### Equipment

The following equipment was used in a typical seed sowing operation using the VSSM, a desktop vacuum source, an Erlenmeyer flask, plastic tubing, 0.8% agar plates containing 0.5 LS salts, sterile filter paper and 95% ethanol (EtOH). The VSSM was fabricated using a Viper Si2 SLA system (3D Systems, Inc., Rock Hill, SC).

### Protocol

**Figure 6:**
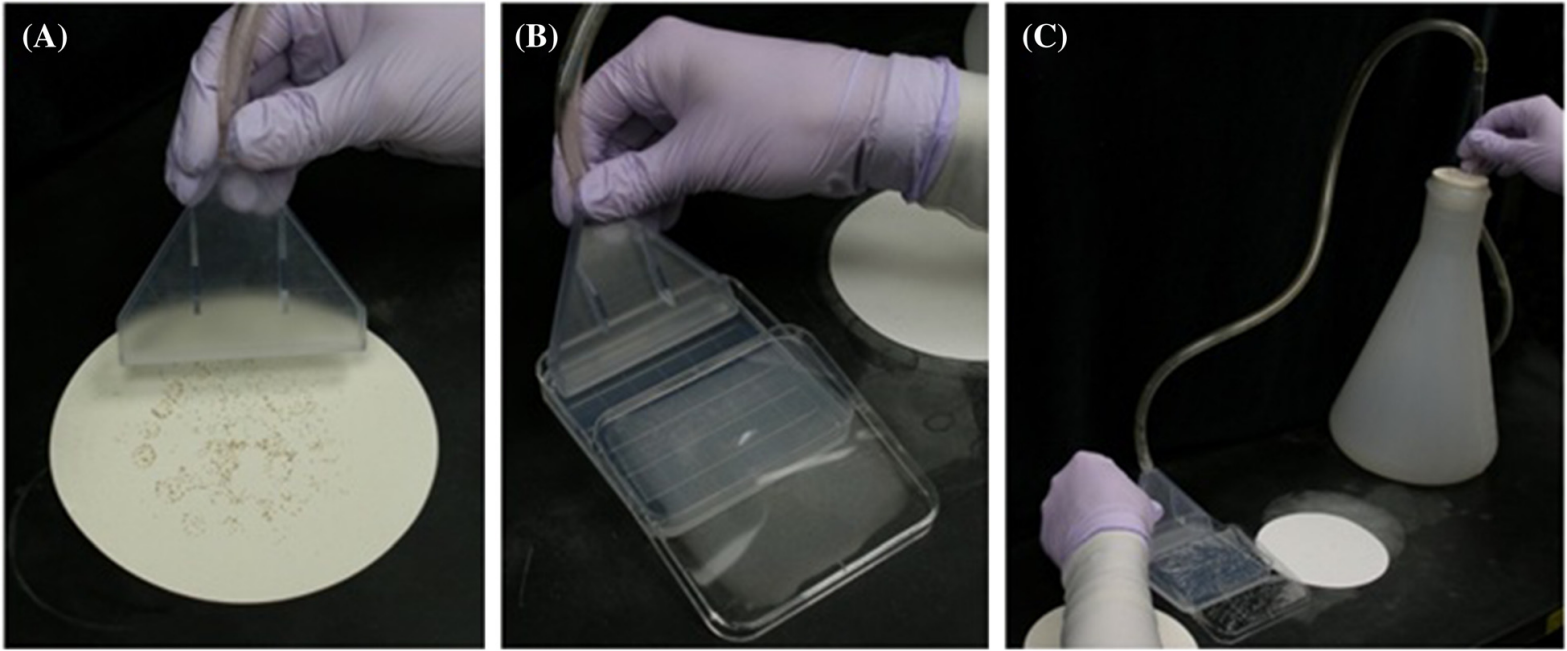
**Seed sowing manifold setup. (A)** Sterile seeds scattered on filter paper are attached to the VSSM by applying a vacuum and touching the holes to the seeds. **(B)** The lid is removed from the agar plate and the VSSM is placed at a 45° angle on the surface of the agar. **(C)** The vacuum is removed and the tip of the VSSM is rotated over the surface of the agar depositing a line of seeds.

Through several trials and seed sowing operations, a general protocol has been developed for using the seed sowing manifold. It is as follows:

1. Select seeds that are at least 0.25 mm in diameter using a 0.25 mm fine sieve.

2. Sterilize surface contamination of the selected seeds in ethanol (EtOH) by rinsing gently at least four times.

3. Spread out the seeds on filter paper to allow the EtOH to evaporate. If the seeds clump together on the filter paper, use a sterile paint brush to spread them out evenly.

4. Attach the SSM adapter to the vacuum source and sterilize it by passing 20% bleach through the small holes at the bottom using an applied vacuum (Figure 6). Make sure all of the holes are submerged during this process, so it is thoroughly sterilized. Some of the holes may be filled with plastic depending on the quality of the manufacturing process. Printing multiple SSM adapters is advisable, to account for corrosion from the bleach and/or blocked holes. Then rinse 3–5 times with sterile water and allow any residual water to evaporate.

5. Apply a vacuum to the device again and place the end of the manifold into the seeds and move it around until many are stuck to the device. They should be much easier to remove than those retained at each hole by the applied vacuum. Repeat this process until nearly all of the holes have a seed on them.

6. Remove any seeds unattached to a hole using the sterile paint brush or by rubbing against filter paper. Be careful to not accidentally knock off a seed that is on one of the holes.

7. Place the sowing adapter on the agar plate (for these initial experiments, 0.8 percent agar was used) and turn off the applied vacuum. Deposit the seeds on the plate by gently rolling it across the surface of the agar. Seeds can be spread out if needed using the sterile paint brush or tooth pick.

8. Now one row of seeds has been successfully sown. Repeat this protocol for each additional line of seeds until the desired number of seeds has been sown.

## Abbreviations

VSSM: Vacuum seed sowing manifold; SSM: Seed sowing manifold; CAD: Computer-aided design; SS: Seed sowing; 3D: Three-dimensional; EtOH: Ethanol; iTRAQ: Isobaric tags for relative and absolute quantitation.

## Competing interests

The authors declare that they have no competing interests.

## Authors’ contributions

RB realized the need for a high-throughput device. RB and BC designed the device and the previous iterations of it. RB and BC prepared the original manuscript. BC fabricated the device using 3D printing technology. RB tested the device by using it to sow seeds for various experiments. TM supervised the fabrication of the device. PM supervised the design of experiments using the device. RB, BC, TM, and PM revised the manuscript critically for important intellectual content and gave final approval of the version to be published. All authors read and approved the final manuscript.

## Authors’ information

RB is a post doctoral research associate in the Genetics department of the College of Agriculture and Life Sciences at the University of Wisconsin at Madison.

BC is an assistant engineer in the Medical Devices Laboratory at Morgridge Institute for Research in Madison, WI and doctoral candidate in the Medical Physics Department at the University of Wisconsin at Madison.

TM is the director of the Medical Devices Laboratory at Morgridge Institute for Research in Madison, WI and a professor emeritus in the Medical Physics Department at the University of Wisconsin at Madison.

PM is a Professor in the Genetics department of the College of Agriculture and Life Sciences at the University of Wisconsin at Madison.

## Supplementary Material

Additional file 1 Video of a researcher using the VSSM to sow a single row of Arabidopsis seedlings. Click here for file

Additional file 2 **Computer-aided design (CAD) file of the VSSM with 50 holes in it.** This is available in two file formats. The first format was generated in SolidWorks (.SLDPRT) and can be edited in SolidWorks or another CAD software for additional or fewer holes. The second format (.STL) is the file type most frequently used by 3D printers and can be used for printing immediately upon download. Click here for file

## References

[B1] Arabidopsis Genome Initiative Analysis of the genome sequence of the flowering plant Arabidopsis thaliana Nature 2000 408 796 815 10.1038/35048692 11130711

[B2] Parkin IA Gulden SM Sharpe AG Lukens L Trick M Olborn TC Lydiate DJ Segmental structure of the brassica napus genome based on comparative analysis with Arabidopsis thaliana Genetics 2005 171 765 781 10.1534/genetics.105.042093 16020789 PMC1456786

[B3] Cao J Schneeberger K Ossowski S Günther T Bender S Fitz J Koenig D Lanz C Stegle O Lippert C Wang X Ott F Müller J Alonso-Blanco C Borgwardt K Schmid KJ Weigel D Whole-genome sequencing of multiple Arabidopsis thaliana populations Nat Genet 2011 43 956 963 10.1038/ng.911 21874002

[B4] Sessions A Burke E Presting G Aux G McElver J Patton D Dietrich B Ho P Bacwaden J Ko C Clarke JD Cotton D Bullis D Snell J Miguel T Hutchison D Kimmerly B Mitzel T Katagiri F Glazebrook J Law M Goff SA A high-throughput Arabidopsis reverse genetics system Plant Cell Online 2002 14 2985 2994 10.1105/tpc.004630 PMC151197 12468722

[B5] Wang Z Gerstein M Snyder M RNA-Seq: a revolutionary tool for transcriptomics Nat Rev Genet 2009 10 57 63 10.1038/nrg2484 19015660 PMC2949280

[B6] Schmidt D Wilson MD Spyrou C Brown GD Hadfield J Odom DT ChIP-seq: using high-throughput sequencing to discover protein–DNA interactions Methods 2009 48 240 248 10.1016/j.ymeth.2009.03.001 19275939 PMC4052679

[B7] Wiese S Reidegeld KA Meyer HE Warscheid B Protein labeling by iTRAQ: a new tool for quantitative mass spectrometry in proteome research Proteomics 2007 7 340 350 10.1002/pmic.200600422 17177251

[B8] Kai CC Fai LK Chu-Sing L Rapid prototyping: principles and applications in manufacturing 2003 Singapore: World Scientific Publishing Co., Inc

[B9] Kai CC Three-dimensional rapid prototyping technologies and key development areas Comput Contr Eng J 1994 5 200 206 10.1049/cce:19940407

[B10] Webb P A review of rapid prototyping (RP) techniques in the medical and biomedical sector J Med Eng Technol 2000 24 149 453 10.1080/03091900050163427 11105287

